# Changes in Triterpenes in *Alismatis rhizoma* after Processing Based on Targeted Metabolomics Using UHPLC-QTOF-MS/MS

**DOI:** 10.3390/molecules27010185

**Published:** 2021-12-29

**Authors:** Mengxiang Dai, Sen Li, Qingxin Shi, Xingliang Xiang, Yuehui Jin, Sha Wei, Lijun Zhang, Min Yang, Chengwu Song, Rongzeng Huang, Shuna Jin

**Affiliations:** 1 College of Pharmacy, Hubei University of Chinese Medicine, 16 Huangjiahu West Road, Wuhan 430065, China; dmx199704@163.com (M.D.); qingxinshi123@hotmail.com (Q.S.); xxl741805629@163.com (X.X.); jinyuehui21@outlook.com (Y.J.); songchengwu2000@163.com (C.S.); 2 Department of Pharmacy, Union Hospital, Tongji Medical College, Huazhong University of Science and Technology, Wuhan 430030, China; lisentome728@hotmail.com; 3 College of Basic Medicine, Hubei University of Chinese Medicine, 16 Huangjiahu West Road, Wuhan 430065, China; weisha2021@hbtcm.edu.cn (S.W.); zhanglijun2021@hbtcm.edu.cn (L.Z.); yangmin2021@hbtcm.edu.cn (M.Y.)

**Keywords:** *Alismatis rhizoma*, triterpenes, processing, multivariate statistical analysis, metabolomics

## Abstract

*Alismatis rhizoma* (AR) has been used as an herbal medicine in China for over a thousand years. Crude AR, salt-processed AR (SAR), and bran-processed AR (BAR) are recorded in the Pharmacopoeia of the People′s Republic of China. However, the differences of chemical composition between crude AR and its processing products remain limited. In this study, triterpenes were identified from crude AR, SAR, and BAR by ultra-high performance liquid chromatography coupled with quadrupole time-of-flight-mass spectrometer (UHPLC-QTOF-MS/MS). Subsequently, the differences of triterpenes between the crude AR and processed ARs were compared via a targeted metabolomics approach. Finally, a total of 114 triterpenes were identified, of which 83, 100, and 103 triterpenes were found in crude AR, SAR, and BAR, respectively. After salt-processing, there were 17 triterpenes newly generated, 7 triterpenes with trends of increasing, and 37 triterpenes decreased. Meanwhile, 56 triterpenes including 21 newly generated and 35 with significant increases were observed in BAR. This study could be benefit to investigate the processing mechanism of AR, as well as support their clinical applications.

## 1. Introduction

In Traditional Chinese Medicine (TCM), processing (“Paozhi” in Chinese) refers to certain types of pharmaceutical technology that promote the use of Chinese herbal medicines to fulfil specific requirements of therapy [1]. Under the guidance of the TCM theory, crude herbs are processed to improve the herb’s efficacy and reduce the herb’s toxicity and side effects using salt, rice wine, or bran, etc. [2]. Accumulating evidence has shown that along with the contents and structures of constituent’s changes, the pharmacological activity of a certain herb usually changes accordingly [2,3]. Thus, the investigation of the changes in the herb’s chemical composition after processing is vital to understand the pharmacological mechanism of the herb and its clinical significance.

*Alismatis rhizoma* (AR, “Zexie” in Chinese), recorded in Chinese Pharmacopoeia (2020 edition), is the dried rhizome of *Alisma orientale* Juzepzuk, belonging to family Alismataceae [4]. It has been used as a TCM for a long time to treat various diseases such as dysuria, edema, nephropathy, hypertension, and hyperlipidemia [5,6,7]. Two processed products of AR, salt-processed AR (SAR), and bran-processed AR (BAR), are also recorded in the Chinese Pharmacopoeia Commission [8]. They were usually processed with salt or bran and heated with a certain temperature until their surface appear yellow [9]. Processed AR with different methods demonstrates different pharmacological effects in the clinic. Previous studies demonstrated that SAR had stronger diuretic activity than AR, while BAR added the function of tonify the spleen [10]. However, the mechanism for the difference in their efficacy is still unclear.

Pharmacological studies have revealed that triterpenes are the main bioactive constituents responsible for the diuretic, anti-hyperlipidemia, antidiabetic, and anti-inflammatory efficacies of AR [11,12,13,14]. A previous study reported that the discrimination of eight triterpenes in AR from different regions [15]. Moreover, recent studies revealed the correlation between the contents of six major triterpenes and the diuretic effect of AR and SAR [16]. However, up to now, quite a few studies have systematically elucidated the influence of different processing methods in the triterpenes between AR, SAR, and BAR. Therefore, exploring the difference in triterpenes between crude AR and processed AR is a prerequisite for understanding their pharmacological mechanisms and clinical significance.

In this study, ultra-high performance liquid chromatography coupled with quadrupole time-of-flight-mass spectrometer (UHPLC-QTOF-MS/MS) was performed to analyze the triterpenes of AR, SAR, and BAR. A targeted metabolomics approach was established to evaluate the changes in triterpenes after processing. Further, the processing mechanism of AR were explored.

## 2. Results

### 2.1. Qualitative Analysis of Triterpenes in AR and Processed AR

**Figure 1:**
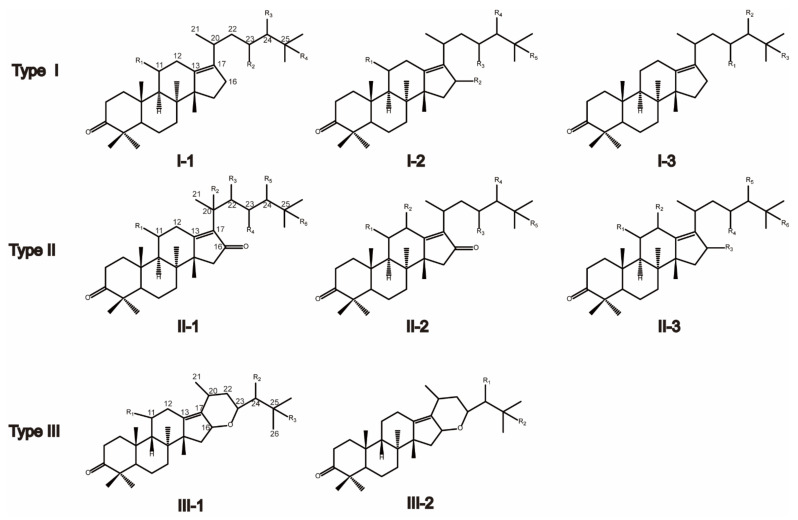
The major triterpene types in *Alismatis rhizoma*.

The classification of triterpenes was performed according to their structural characteristics and former references. As shown in Figure 1, triterpenes mainly differed in their prevalent oxygenation pattern at the position C_16_. Type I and type II triterpenes had open aliphatic chains at C_17_, while the type III triterpene had a six-membered ring system with an oxygen bridge between C_16_ and C_23_. In contrast to type I triterpenes, type II triterpenes possessed keto groups at C_16_ in their structure. According to the differences of substitute position, these three types of triterpenes were further divided into eight sub-types. For instance, the type I-1 triterpene had a hydroxyl or acetyl group substitute at C_11_. The type I-2 triterpene was similar to the type I-1 except for the substitution of C_16_. Furthermore, type I-3 triterpenes had no substituent at position 11. Similarly, the differences among the other sub-types of triterpenes can be ascribed to the presence or absence of substituent groups at C_11_, C_12_, and C_16_.

According to the different structural types, the collision-induced dissociation fragmentation patterns for representative triterpenes of each type skeletons were shown in Figures S1–S8. Based on the type of triterpenes and corresponding fragmentation rules, the triterpenes of the same type could be identified. Triterpenes with a diagnostic product ion at *m*/*z* 339.26 could be identified as type I-1 triterpenes. Type I-2 and I-3 triterpenes exhibited a typical ion at *m*/*z* 337.25 and 341.28, respectively. In addition, product ion at *m*/*z* 353.25, 369.24, and 355.26 were the characteristic ions of type II-1, II-2, and II-3 triterpenes, respectively. In addition, *m*/*z* 339.27 and 381.28 were used as the diagnostic product ions of type III-1 triterpenes, whereas type III-2 triterpenes exhibited diagnostic product ions at *m*/*z* 341.28 and 383.29. For instance, alisol A 24-acetate was a representative compound of type I-1, and its proposed fragmentation pathway was shown in Figure S1. Alisol A 24-acetate had a [M + H − H_2_O]^+^ ion at *m*/*z* 515.3700 in the full mass spectra scans. The product ions at *m*/*z* 497.35, *m*/*z* 455.35, and *m*/*z* 419.33 were formed by successive loss of H_2_O and AcOH. The *m*/*z* 383.29 ion was observed in the product ion related to the dissociation of the C_23_–C_24_ bond, and the product ion at *m*/*z* 365.28 was acquired by a further loss of H_2_O from the ion of *m*/*z* 383.29. In addition, the characteristic fragment at *m*/*z* 339.26 was obtained by losing C_2_H_2_ from *m*/*z* 365.28. Therefore, the product ion at *m*/*z* 339.26 can be used as characteristic diagnostic ions for screening the same type of triterpenes. Similarly, the other types of triterpenes could be identified based on the fragmentation rules and corresponding characteristic product ions.

**Figure 2:**
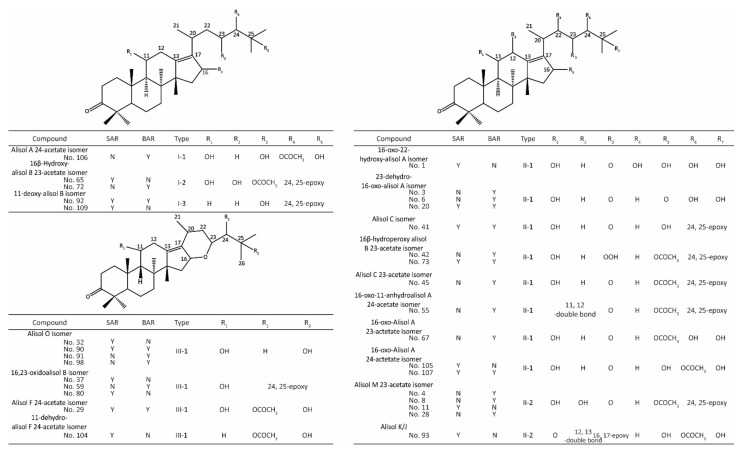
The new generated triterpenes in salt and bran processed AR. N: The compound was not detected in the sample. Y: The compound was detected in the sample.

Finally, a total of 114 triterpenes (83, 100, and 103 compounds in AR, SAR, and BAR, respectively) were identified in AR. Among these triterpenes, 10 were type I-1 triterpenes, 8 were type I-2 triterpenes, 6 were type I-3 triterpenes, 43 were type II-1 triterpenes, 15 were type II-2 triterpenes, 6 were type II-3 triterpenes, 22 were type III-1 triterpenes, and 4 were type III-2 triterpenes. In addition, the results indicated that 17 new triterpenes were discovered in SAR (Figure 2), including compound **1**, **11**, **20**, **29**, **32**, **37**, **41**, **65**, **73**, **80**, **90**, **92**, **93**, **104**, **105**, **107**, and **109**. As for BAR, 21 new triterpenes were found in BAR (Figure 2), including compound **3**, **4**, **6**, **8**, **20**, **28**, **29**, **41**, **42**, **45**, **55**, **59**, **67**, **72**, **73**, **90**, **91**, **92**, **98**, **106**, and **107**. Meanwhile, compound **113** which was identified in crude AR disappeared in BAR. The details of the identified triterpenes were summarized in Table S1 and their structures were shown in Table S2.

### 2.2. Targeted Metabolomic Analysis of Triterpenes

**Figure 3:**
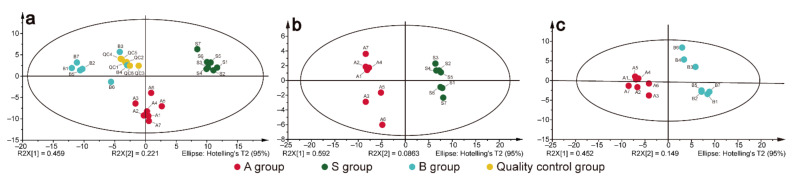
Multivariate statistical analysis of metabolic profiles derived from various AR samples: (**a**) PCA score scatter plots obtained from the crude samples (group A), salt-processing samples (group S), bran-processing samples (group B) and quality control samples (group QC). (**b**) PLS-DA score scatter plots obtained from A and S group. (**c**) PLS-DA score scatter plots obtained from A and B group.

A targeted metabolomic analysis was used to compare the triterpenes between AR and processed ARs. In the unsupervised principal component analysis (PCA) model (Figure 3a), the separation was significant (R^2^X = 0.73, Q^2^ = 0.666), which indicated a difference in the levels of triterpenes between crude AR and processed ARs. Thus, the results indicated that different changes in triterpenes had occurred after processing compared with crude AR. The supervised partial least squares-discrimination analyses (PLS-DA) was used to further explore the differences between crude and processed ARs.

As shown in Figure 3b,c, the PLS-DA score plots illustrated that the A group was clearly separated from the S and B groups. The results of PLS-DA models showed that values of R^2^Y and Q^2^ were 0.99 and 0.976 for A group and S group, and 0.993 and 0.953 for A group and B group, respectively, which indicates that the models had good abilities for both prediction and reliability. Moreover, the Q^2^ of the 999-time permutation tests of the aforementioned models were both negative, indicating the two PLS-DA models were not overfitting (Figure S9).

**Figure 4:**
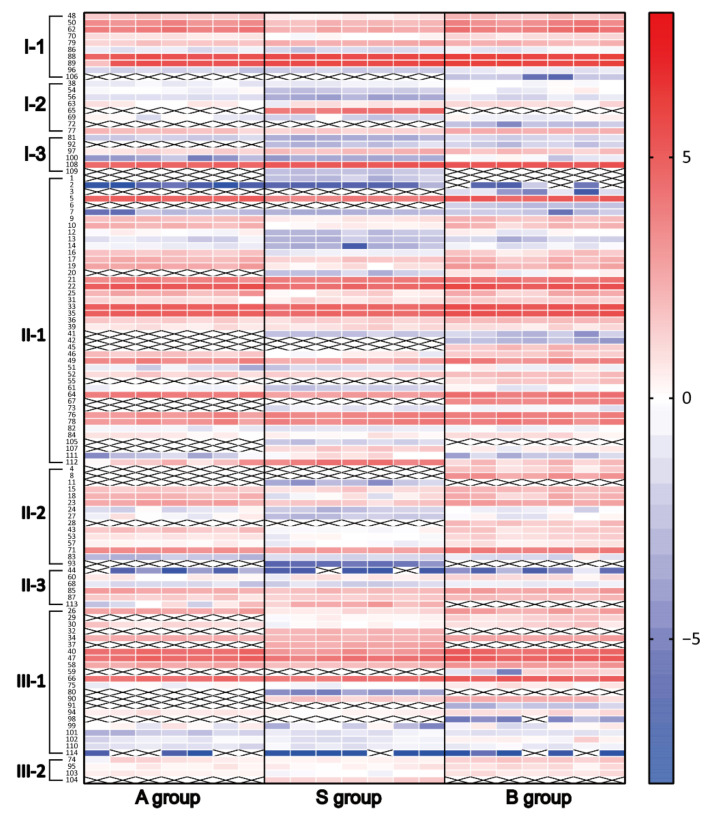
The relative contents of triterpenes detected in the extracts of three samples. A, S, and B are the crude samples, salt-processed samples, and bran-processed samples, respectively. The value of each compound was the log2 transformation of its relative content. The color intensity from blue to red reflects the relative content of each triterpenes. The triterpenes not detected in the sample were marked as cross in the heatmap.

To improve the visualization, these profiles were displayed as heatmap (Figure 4). As depicted in Figure 4, it was observed that most of triterpenes had lower relative contents in the group S, while there were no significant differences in the color of the same triterpenes between group A and group B. Some triterpenes were newly generated after processing, for example, compound **1**, **3**, and **11**. The heatmap results provided an overview of the qualitative changes in triterpenes and their variations in relative contents between crude AR and processed ARs. 

### 2.3. Discovery the Changes in Triterpenes in SAR and BAR

**Figure 5:**
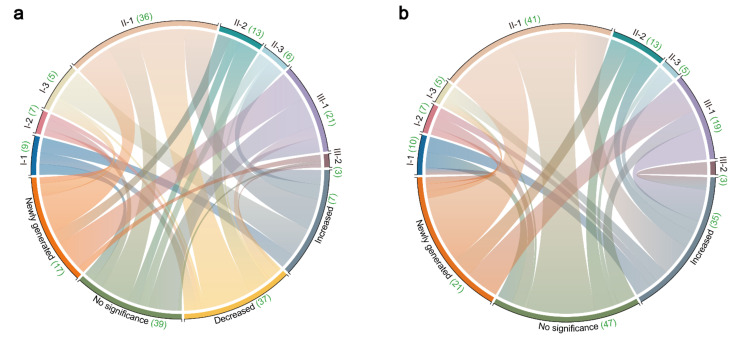
Distribution of triterpenes in the extracts of two processing groups: (**a**) salt-processing samples. (**b**) bran-processing samples. The number of different types of triterpenes is indicated by the green number.

As depicted in Figure 5, the triterpenes were divided into four groups, including newly generated group, no significance group, decreased group and increased group. According to the PLS-DA analysis (VIP > 1.0) and one-way ANOVA test (*p* value < 0.05), 44 triterpenes occurred significant differences in SAR, including 37 decreased and 7 increased triterpenes. Meanwhile, 35 triterpenes were found to increase significantly in BAR and there were no significant differences in other triterpenes. Compared to crude AR, there were 17 triterpenes newly generated in SAR. The majority of them were type II-1 and type III-1 triterpenes, accounting for 35.29 and 29.41%, respectively. As for BAR, 21 triterpenes newly generated and more than half of them were type II-1 triterpenes (accounting for 61.90%). 

**Figure 6:**
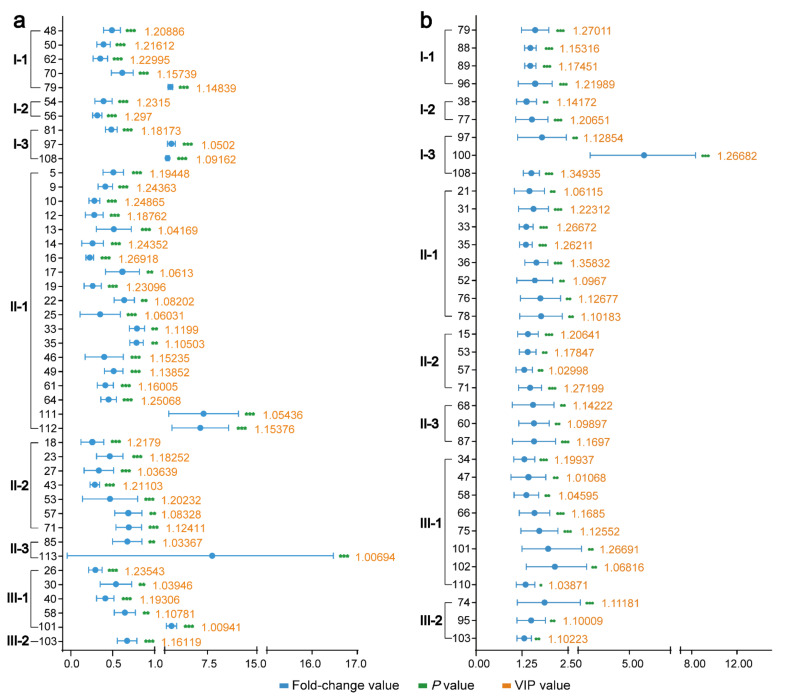
Triterpenes with significant differences between different AR samples: (**a**) Distribution of triterpenes with significant differences between crude and salt-processed sample based on different structure. (**b**) Distribution of triterpenes with significant differences between crude and bran-processed sample based on different structure. Values at each point was the ratio of the mean content of a triterpene in processed AR to that of crude AR. Error bars represent the mean ± S.D. Statistics were performed with one-way ANOVA test, indicated as * *p* value < 0.05, ** *p* value < 0.01 and *** *p* value < 0.001. The orange number represents the VIP value of the triterpenes.

Further, the triterpenes with significant differences between AR and its processed products were illustrated as the error line diagram of fold changes based on eight types (see Figure 6). The fold change in a triterpene was the ratio of the mean content of a triterpene in processed AR to that of crude AR. Thus, a fold change value > 1.0 meant an increased content of the related triterpene after processing, whereas a fold change value < 1.0 meant a decreased content. It was obvious that all 35 triterpenes with significant differences had an increased in content after bran-processing, while in SAR group, significantly changed triterpenes were mainly decreased in content.

## 3. Discussion

To explore the differences between crude and processed ARs, the UHPLC-QTOF-MS/MS combined with multivariate statistical analysis was developed to identify and distinguish the difference in triterpenes in crude AR and processed ARs. In this study, a total of 83, 100, and 103 triterpenes were identified in crude AR, SAR, and BAR, respectively. There were 17 and 21 newly generated triterpenes were detected in SAR and BAR. Meanwhile, compared with the content of triterpenes in crude AR, 44 triterpenes had significantly changed after salt-processing, including 37 decreased and 7 increased triterpenes. While after bran-processing, 35 triterpenes were significant differences and all of them showed an increase in contents.

Many methods of processing, such as stir-frying, steaming, and boiling, need to be carried out at the heating conditions, which probably leads to complex chemical changes [17]. In this study, AR was processed with salt water under the temperature range of 80 to 120 °C. Our results showed that in SAR, compounds **14**, **17**, **18**, **19**, **22**, **23**, **25**, **27**, **33**, **35**, **43**, **46**, **49**, **53**, **57**, **64**, **71**, and **75**, which all possessed a C_24_, C_25_-oxide three-membered ring of the side chain were massively reduced in type II triterpenes compared to type I triterpenes. It was speculated that the type II triterpenes with a C_24_, C_25_-oxide three-membered ring of the side chain were more unstable than others. A previous study indicated that owing to the presence of a carbonyl group at C_16_, cracking and ring opening of triterpenes at position 24 and 25 was more easily than others [18]. In addition, the contents of type I (compound **48**, **50**, **54**, **56**, **62**, **70**, and **81**) and type III (compound **26**, **30**, **40**, **58**, and **103**) triterpenes with hydroxyl or acetyl groups at C_23_, C_24_, and C_25_ were significantly reduced after salt-processing. It was reported that the compounds with substituting groups, such as hydroxyl and acetyl groups, would lose H_2_O and AcOH under high temperature [16,19]. Moreover, there was the evidence suggesting that the total contents of triterpenes were decreased with the baking temperature increasing, especially when the temperature was above 80 °C [20]. 

However, the reduction in certain triterpenes after heating was not observed in BAR, on the contrary, triterpenes with significant differences showed an increasing trend after bran-processing. Our findings indicated that triterpenes in crude AR had different change trends after two processing of SAR and BAR. In this study, AR was processed at two methods with different excipients (salt and bran), temperature ranges (80 to 120 °C and 150 to 220 °C) and heating durations (around 30–40 min and around 8 min); thus, these parameters might be responsible for the different change trends of triterpenes between SAR and BAR. In addition to the heating temperature, heating duration was also an important factor in AR processing. A report about triterpenes suggested that the contents of alisol A 24-acetate and alisol B 23-acetate initially increased as the duration of heating time, but after 40 min of heating, this increasing tendency ceased and the concentrations rapidly decreased [21]. Considering the heating duration of around 8 min for bran-processing in our study, the increase in triterpene contents in BAR were consistent with the previous report. Meanwhile, emerging evidence has indicated that under heating condition, excipients used in processing could often help active constituents to dissolve more easily from a complex texture [22,23]. The existing of bran made sample heated evenly, and the heat duration of BAR was much shorter than SAR. Taken together, the current study demonstrated that the heating temperature, heating duration, and excipient were the crucial factors in AR processing. 

For the effectiveness of TCM, herbal may profit from synergistic functions of multiple chemical ingredients [24,25]. Although most of triterpene’s contents were decreased, there were some triterpene contents that increased and newly formed after salt-processing. For example, alisol A (compound **79**), 11-deoxy-alisol B (compound **97**), 11, 25-anhydro-alisol F (compound **101**), and 11-deoxy-alisol B 23 acetate (compound **108**) were found with significance increases after processed with salt. In addition, the therapeutic efficacy of SAR might benefit from synergistic actions of multiple ingredients. During the processing, complicated changes in herbal components may occur, and the pharmacological activity of a certain herbal medicine may be changed accordingly [1]. Previous studies demonstrated that SAR showed a better hepatoprotective activity and diuretic effect than crude AR [16,26]. This phenomenon also occurs in other salt-processed plant medicine [27,28].

Generally, the main purposes of processing are reducing toxicity and enhancing the effects of crude drugs. Previous studies indicated that the hydroxyl and acetyl groups on the side chain of triterpene were the essential groups to exert lipid-lowering effect [29,30]. For example, alisol A (compound **79**) is one of the main active triterpenes in AR and possesses many biological activities including antibacterial, antiviral, lipid regulating, and diuretic effects [16,31,32,33]. It showed a similarity in the pattern of content changes in SAR and BAR (the fold change value of SAR and BAR was 1.849 and 1.566, respectively). Furthermore, it was reported that triterpenes with the carbonylation on the C_16_ of the parent nucleus and a C_24_, C_25_-oxide three-membered ring of the side chain had relation with the lipid-lowering activity [9]. In this study, among the newly generated and increased triterpenes, most of them showed similar characteristic features, which were a C_24_, C_25_-oxide three-membered ring of the side chain no matter in SAR or BAR. Moreover, processing Chinese medicine can enhance the efficacy and change the trend of action [1,34]. The difference in changing trends of triterpene’s contents between SAR and BAR might bring out potential changes in pharmacological activity. Since salt-processing could enhance the effect of AR in diuretic activity [16], the contents increased in BAR might reinforce the activity of AR. Results of a previous study indicated that the diuretic effect of BAR was better than SAR [18]. In addition, there was the evidence showed that SAR exhibited better hepatoprotective effects than crude and bran-processed AR by lowering levels of serum AST and ALT [35]. In conclusion, the effects of heating, heating durations, and excipients in the processing procedure might be the main causes of the changes in chemical composition, as well as the subsequent impacts on the efficacy of crude AR.

## 4. Materials and Methods

### 4.1. Chemicals and Reagents

Acetonitrile (ACN, LC-MS grade) and formic acid (≥98%, analytical grade) were purchased from Fisher Scientific (Fair Lawn, NJ, USA) and Sinopharm Chemical Reagent Co., Ltd. (Shanghai, China), respectively. Purified water was obtained from Millipore using a Milli-Q system (Bedford, MA, USA). The dried rhizomes of cultivated AR were collected from Sichuan province of China in April 2019 and identified by Prof. Keli Chen of Hubei University of Chinese Medicine. The dried rhizomes of AR were cut into 2–4 mm slices before used. 

The reference standards (alisol F, alisol F-24-acetate, 24-deacetyl alisol O, alisol A 24-acetate, alisol B 23-acetate and alisol C 23-acetate) were previously isolated and purified from the dried rhizome of AR. The purities of these standards were tested to be above 98% using HPLC coupled with UV detector. 

### 4.2. Preparation of SAR and BAR

SAR and BAR were prepared according to the procedure recorded in the general principles of Chinese pharmacopoeia (2020 edition) [36]. Briefly, the crude AR slices (1 kg) was mixed and soaked with 2% salt solution (2:100, salt-water, *w*/*v*) until fully absorbed in a sealed container. Then the moistened AR were stir-fried in a metallic pan over a low flame (80–120 °C) around 30–40 min until the water evaporated. Similarly, the bran (100 g) was placed in a preheated stir-frying pan. When the bran was fuming, added 1 kg of AR and stirred rapidly around 8 min until the color of AR turned yellow, and removed the bran. After cooling to room temperature, SAR and BAR were obtained and used for further analysis.

### 4.3. Preparation of Sample Solutions

Seven batches of AR were smashed and sieved (24 mesh) to obtain homogeneous powders separately. Sample powder (0.5 g) was ultrasonically extracted with 5 mL acetonitrile at room temperature for 30 min. Then, the supernatant of these samples was centrifuged at 14,000 rpm for 10 min followed by filtration, after which 5 μL of supernatant of each sample with fexofenadine (100 ng·mL^−1^) as internal standard was injected and analyzed by the UHPLC-QTOF-MS/MS. SAR and BAR were extracted with the same procedure as above. Thus, the samples were separated into 3 groups: raw AR group (A group), salt-processed AR group (S group), and bran-processed AR group (B group). Meanwhile, the quality control samples (QC) were prepared by pooling each solution from all samples. To assess of the stability of analytical systems, the QC samples were performed at the beginning, the end, and every 5 injections throughout the whole UHPLC-MS/MS analysis.

### 4.4. Chromatography and Mass Spectrometric Analysis

UHPLC-MS/MS analysis was performed on a Waters ACQUITY Ultra Performance LC system. A C18 column (100 mm × 2.1 mm i.d., 1.7 μm, Waters, Milford, MA, USA) was used for separation. The flow rate was 0.3 mL·min^−1^ and the injection volume was 5 μL with a column temperature at 40 °C. The mobile phase of the eluent was 0.1% formic acid in water (A) and acetonitrile (B), and the gradient conditions were as follows: 0–18 min, 20–77% B; 18–25 min, 77–95% B; 25–27 min, 95% B; 27–28 min, 95–20% B; 28–30 min, 20% B.

Mass spectrometric analyses were performed on a Waters Xevo G2-XS QTof system with an electrospray ionization source and the positive ion mode was used for data acquisition. The following MS conditions were set: cone gas flow, 50 L·h^−1^; desolvation gas flow, 600 L·h^−1^; desolvation temperature, 500 °C; source temperature, 100 °C; cone voltage, 60 V; capillary voltage, 3 kV. The collision energy was set at 30 eV and the collision energy spread was 40 eV for MS/MS experiments. The mass ranges were set at *m*/*z* 100–1200 for full scan with a scan duration of 1 sec.

### 4.5. Statistics Analysis

The raw spectra data were acquired and managed using MassLynx software (Waters Corporation, Milford, MA, USA). Quantitative results were obtained by dividing the peak area of each triterpene by the peak area of the internal standard fexofenadine. After this procedure, the resulting dataset was on multivariate statistical analysis and one-way ANOVA using the SIMCA-P software (Umetrics AB, Umea, Sweden, version 14.1) and GraphPad Prism (GraphPad Software, La Jolla, CA, USA).

To evaluate the differentiation of chemical compounds caused by different processing methods, crude AR, SAR, and BAR were set as groups A, S, and B, respectively. QC samples were set as group QC and used to evaluate the repeatability and stability of the whole sample set [37]. To determine whether the triterpenes in extract of processed AR differed from those of the crude AR, PCA was used for multivariate exploration of clusters and trends among the four groups. Subsequently, using PLS-DA, an examination for the differences of triterpenes between AR samples was achieved. Moreover, permutation tests (999 cycles) were conducted to assess model overfitting. The cross-validation parameters R^2^Y and Q^2^ were calculated to evaluate the goodness-of-fit and predictive ability of PLS-DA model. Variable importance in the projection (VIP) was a parameter that showed the importance of a variable in a model. Usually, a compound with VIP > 1.0 was considered to play an important role in distinguishing groups [37,38]. Triterpenes that met the following two criteria were regarded as the differential metabolites between crude AR and process ARs. First, VIP values in PLS-DA model were greater than 1.0. The other was that the contents of triterpenes had significant differences (*p* value < 0.05) calculated by one-way ANOVA.

## 5. Conclusions

In this study, UHPLC-QTOF-MS/MS combined with a targeted metabolomics analysis was applied to investigate the changes in triterpenes between crude AR and processed ARs. As a result, a total of 83, 100, and 103 triterpenes were identified in crude AR, SAR, and BAR, respectively. Except for the newly generated triterpenes, there were 44 and 35 triterpenes were changed significantly after salt and bran processing. Our results suggested that triterpenes in crude AR had different change trends after two processing of SAR and BAR. In SAR group, significantly changed triterpenes were mainly decreased in content, while all 35 triterpenes with significant differences had an increased in content after bran-processing. The different change trends between SAR and BAR could be attributed to different heating durations, temperatures, and excipients. The results of this study could provide scientific evidences for the variations of triterpenes in AR under different processing methods, and be valuable to elucidate the processing mechanisms of AR. Further studies will focus on the relationship between the differences of triterpene and pharmacological activity after AR processing.

## Data Availability

The datasets generated and/or analyzed during the current study are available from the corresponding author on reasonable request.

## Supplementary Materials

The following supporting information can be downloaded online. Figure S1: The proposed fragmentation pathway of alisol A 24-acetate, Figure S2: The proposed fragmentation pathway of 16β-Hydroxy-alisol B 23-acetate, Figure S3: The proposed fragmentation pathway of 11-deoxy-alisol B, Figure S4: The proposed fragmentation pathway of alisol C 23-acetate, Figure S5: The proposed fragmentation pathway of alisol M 23-acetate, Figure S6: The proposed fragmentation pathway of 11-Deoxy-alisol C 23-acetate, Figure S7: The proposed fragmentation pathway of alisol O, Figure S8: The proposed fragmentation pathway of alisol I, Figure S9: Goodness of fit and validations (permutation tests) of the PLS-DA models. (a) scoring plots for 999-time permutation validation test for PLS-DA models that corresponded to the raw sample and salt-processed sample. (b) scoring plots for 999-time permutation validation test for PLS-DA models that corresponded to the raw sample and bran-processed sample, Table S1: Identification of triterpenes obtained from the extract of *Alismatis rhizoma* using UHPLC-QTOF-MS/MS, Table S2: Chemical structures of identified triterpenes from *Alismatis rhizoma.*

## Sample Availability

Samples of the crude *Alismatis rhizoma*, salt-processed *Alismatis rhizoma* and bran-processed *Alismatis rhizoma* are available from the authors.

## Abbreviations

TCM	traditional Chinese Medicine;
AR	*Alismatis rhizoma*;
SAR	salt-processed AR;
BAR	bran-processed AR;
UHPLC-QTOF-MS/MS	ultra-high performance liquid chromatography coupled with quadrupole time-of-flight-mass spectrometer;
PCA	principal component analysis;
PLS-DA	partial least squares-discrimination analyses;
VIP	variable importance in the projection;
One-way ANOVA	one-way analysis of variance;
A group	raw AR group;
S group	salt-processed AR group;
B group	bran-processed AR group;
QC	quality control samples.
